# Exploring Issues of Culture in Peer Work – A Qualitative Study

**DOI:** 10.1007/s10488-026-01509-7

**Published:** 2026-05-23

**Authors:** Niharika Hiremath, Louise Byrne, Helena Roennfeldt, Jonathan P. Edwards, Claudia McKenley, Anton Isaacs, Chyrell D. Bellamy

**Affiliations:** 1 Mind Australia Group, Melbourne, Australia; 2 Solis Culture and Mental Health, Victoria, Australia; 3 Lived Experience Training, Yeppoon, Queensland, Australia; 4 School of Medicine, Department of Psychiatry, Program for Recovery and Community Health, Yale University, New Haven, United States; 5 School of Medicine, Department of Psychiatry, Program for Recovery and Community Health, Yale University, New Haven, United States; 6 School of Social Work, Columbia University, New York, United States; 7 School of Rural Health, Monash University, Warragul, Victoria, Australia

**Keywords:** Peer work, Lived experience, Lived expertise, Culture, Culturally and racially marginalized representation, Workforce development

## Abstract

Peer work and Lived Expertise are vital for shaping individual and collective understandings of mental health, addiction, and behavioral health service systems. Culturally and Racially Marginalized [CARM] people are underrepresented in peer support work but also encounter multiple challenges as they navigate often traditional service settings. There is an urgent need for more diverse voices within these spaces, especially those of CARM communities. This research aims to contribute to a deeper understanding of the complexities surrounding experiences of equity, diversity, inclusion, and belonging by peer workers employed in traditional behavioral health settings. Employing grounded theory methods to guide data collection and analysis, it examined qualitative data derived from a larger study of peer workers from five multidisciplinary organizations in the United States. The analysis identified key themes and challenges encountered by diverse CARM peer workers within behavioral health systems. The themes include: Potential enablers for CARM individuals in peer work contexts; Mental health and AOD systems dominated by white cultural perspectives; CARM people not having a voice in the peer movement; and Challenges faced by CARM people within peer work contexts and when taking on peer work roles. Each domain was divided into subthemes that either support or hinder the promotion of cultural responsiveness in settings that center lived experience and Lived Expertise. The findings of this study have significant implications for practice, underscoring the need for systemic and structural changes to ensure peer work environments are inclusive, equitable, and reflective of the diverse, intersectional communities they serve.

## Introduction

Peer work and Lived Expertise are vital for shaping individual and collective understandings of mental health, addiction, and the behavioral health service systems that are designed to address these issues. Grounded in experiential learning, these approaches offer a deeply human perspective that fosters holistic, empathetic healing and recovery (Byrne & Roennfeldt, 2025; Davidson et al., 2012). In practice, the concepts, values, and principles of peer work are derived from the meanings people attribute to their firsthand experience, forming a unique knowledge base known as Lived Expertise. This collectively built knowledge offers alternative views of adversity and healing that often challenge the perceptions of the mainstream service system and biomedical approaches (Byrne et al., 2016, 2019). A key benefit of this discipline is its role in providing the impetus for transformational change, advocating for recovery-oriented practices, and holding the service system accountable to the broader mental health reform agenda (Clossey et al., 2018).

With the benefits that peer work – and Lived expertise—can bring to the field, there is an urgent need for more diverse voices within these spaces—especially Culturally and Racially Marginalized (CARM) [See Appendix A for Nomenclature] communities. Given that culture has been documented as core to and inseparable from a person’s lived experience, particularly in peer contexts (Hodges et al., 2023), this presents a vital opportunity to ensure our workforces authentically meet the needs of CARM communities. The peer workforce faces unique and complex obstacles in achieving representation. These challenges manifest not only in the underrepresentation of CARM people in peer support work but also in the hurdles they encounter as they navigate often traditional service settings that are also lacking in people who represent CARM perspectives. Specifically, this gap in representation is not merely a matter of numbers; it speaks to the broader need for more inclusive and culturally responsive service models. Existing literature highlights several key challenges faced by CARM people in the peer movement. This includes the lack of intersectional representation within the broader Peer and Madness Movements (LeFrançois et al., 2013) and the dominance of mainstream, White perspectives within behavioral health systems. Addressing these challenges is especially prudent given the “interconnectedness of cultural and social inequalities, shedding light on the danger of focusing only on cultural constructions…rather than also acknowledging the structural factors underpinning mental health” (Mescouto et al., 2024).

Within the broader peer movement, a particularly emphasized issue is the tension between peer culture and mainstream service delivery paradigms. In addition to this identified “culture clash”, CARM peer workers also encounter unequal opportunities in services, face discrimination based on their historical experiences in society, and must contend with stereotypes and stigma relating to lived experience within their own cultures. Leadership structures, within both the broader peer workforce and peer support organizations, tend to skew toward White individuals (Cabassa et al., 2017), further exacerbating the lack of representation. This also speaks to broader structural inequities and barriers faced by diverse peers and workforces (Truong et al., 2014). Representation matters not only for the equitable inclusion of CARM workers within the peer support workforce but also for ensuring that peer workers reflect the diverse backgrounds and experiences of the individuals and communities they serve. The inclusion of CARM workers is especially pertinent given that some communities value shared identity with practitioners as part of their therapeutic journey (Sadusky et al., 2023). Additionally, there is a pressing need for peer support organizations to proactively embrace diversity and foster inclusivity. This is especially salient considering emerging literature proposing that diverse communities can benefit greatly from formal and informal peer support that “interrupts harm and prioritizes agency, consent, and self-determination” (Bakshi, 2021).

Approaches to address these obstacles have included the imperative to increase the representation of individuals from CARM backgrounds within organizational settings, whilst continuing to build broader cultural responsiveness and ongoing relationships with communities (Byrne et al.,  2021). Despite efforts by some organizations, significant workforce challenges persist, including those related to recruiting, retaining, and expanding culturally and ethnically diverse people into various positions within organizations.

Despite growing recognition of these challenges, there remains a dearth of comprehensive literature addressing the specific experiences and needs of Culturally and Racially Marginalized (CARM) people within the peer workforce. This study seeks to address this gap by examining qualitative data derived from a larger study of peer workers in the United States. By identifying and analyzing the challenges faced by CARM people within the peer workforce, this research aims to contribute to a deeper understanding of the complexities surrounding experiences of equity, diversity, inclusion, and belonging by peer workers employed in traditional behavioral health settings. In particular, the analysis of this data may support and inform broader human-centered approaches that seek to expose and transform embedded systems.

## Methods

### Study Design

The parent grant examined factors that support the effective integration of peer roles within mental health service delivery. The present analysis represents a focused qualitative examination of culture-related themes that emerged across the full dataset generated by the parent study (Byrne & Roennfeldt, 2025; Byrne et al., 2021a, 2021b, 2022).

The parent study employed grounded theory methods (Corbin & Strauss, 2015) to guide data collection and analysis. The aim was to explore participants’ understanding of peer roles, including the lived experiences necessary for peer work, factors that make peer workers effective in these positions, and the organizational conditions that support their employment. The primary intent was not to develop a novel theoretical framework but rather to systematically build on and refine existing theory.

### Participating Organizations

A twelve-member expert advisory group of lived-experience leaders, industry representatives, funders, and researchers provided consultation and helped identify multidisciplinary organizations. The advisory group nominated organizations that they believed demonstrated a commitment to effective peer workforce development. The selection of participating organizations was based on the following criteria: clarity of the peer role, support strategies such as supervision and financial sustainability, understanding of peer work among non-peer staff, career mobility for peers, and the organization’s ability to meet diverse needs.

Organizations were contacted for participation, and executive management provided a signed letter confirming their organization’s participation before the study began. Details about the study and an invitation to participate were sent through organizational email lists. All employees could participate by contacting the researcher directly. Recruitment continued until each cohort at every site was represented. Participation was voluntary, and participants gave written consent prior to study commencement.

Five multidisciplinary organizations participated, ranging in size from medium (50–250 employees) to large (more than 250 employees), and included: two not-for-profit, one county-run, one under the umbrella of a managed care company, and one privately owned. The five organizations, anonymized and randomly named Sites 1—5, were located in different parts of the United States (Southeast (Florida), New England (Massachusetts), Southwest (Southern California), Northwest (Northern Oregon) and Northeast (New York)). These sites offered services in diverse settings, including rural, regional, and urban areas. The term multidisciplinary refers to a team of various behavioral health workers, including nurses, psychologists, peer support workers, etc.

### Data Collection

Fourteen (14), etc...

Fourteen (14) focus groups were conducted, and eight (8) individual interviews were conducted. Focus groups were organized for each cohort: non-peer workers, management, and peer-designated employees. Due to smaller numbers, peer managers could choose to attend either the peer worker or management focus group. Two peer managers chose to join the peer worker focus groups, while the rest opted for individual interviews.

Interviews and focus groups began with broad preliminary questions, consistent with grounded theory (Birks & Mills, 2015; Corbin & Strauss, 2015). The questions related to understanding what is essential to peer work, the barriers that exist for peer workers, and strategies for effective recruitment and employment of peer workers in their organization.

In accordance with grounded theory methodology, questions were expanded and refined throughout the interviews and focus groups as participants contributed new concepts, including cultural issues. Data collection continued until conceptual saturation was achieved, at which point no additional insights emerged (Corbin & Strauss, 2015).

There was representation from both peer and non-peer participants at all sites to provide both perspectives. Four of the sites had focus groups for each participant group (designated peer roles, non-designated roles, and management positions), while the remaining site had focus groups for management and non-designated roles, with peer workers at this site choosing to participate in interviews. Four of the sites held interviews and focus groups, with participants at one site participating only in focus groups. The majority of interviews and focus groups were conducted face-to-face, with one interview and one focus group via video call. Focus groups and interviews were audio-recorded and transcribed verbatim. (blind – to be named) University granted IERB approval.

All focus groups lasted 90 min, and interviews ranged from 60 to 90 min. The second author conducted all interviews and focus groups. Neither organizations nor individual participants received any payment. To maintain confidentiality, individual names and organizations were given codes, and city/state locations are not included in reporting.

### Data Analysis

De-identified transcripts were manually coded using QSR International’s NVivo 12 (2018). Researchers applied grounded theory techniques to systematically examine transcripts, develop coding frameworks, and organize those codes into overarching themes (Braun & Clarke, 2006; Corbin & Strauss, 2015). In keeping with grounded theory, data were analysed using a constant comparative approach (Glaser, 2008). The second and third authors conducted the initial analysis, coding the data by identifying key features and documenting emerging patterns and themes, including specific issues participants raised that related to the lack of representation and exclusion in peer work. The remaining authors, who identified as CARM, analyzed and clarified themes relating to the interplay between culture and peer work—such as underrepresentation in the peer movement and the dominance of white culture in mental health systems—which were prominent in the original study. Authors then met to collectively discuss emerging interpretations, refine themes, and reflect on how their cultural backgrounds and systemic contexts shaped the analytical process. This inductive process of theme development led to linking the codes relationally to form key themes and subthemes (Birks & Mills, 2015; Corbin & Strauss, 2015).

## Results

### Study Participants: Demographics

Study participants included 132 individuals: 32 non-peer workers, 47 non-peer managers, 7 peer-designated managers, 38 peer workers, and 8 ‘other’. Participants who chose the ‘other’ category were employed in human resources and administrative roles (e.g., executive assistant). Participants were from various racial/ethnic backgrounds, including 15 identifying as African American or Black, 10 identifying as Multiracial, 7 identifying as Asian, 6 identifying as Hispanic, 2 identifying as Native American, 1 participant identifying as a Native Hawaiian/Pacific Islander, and 88 participants identifying as Caucasian American or White. Participants at each organization were as follows: 31 at Site 1, 27 at Site 2, 31 at Site 3, 23 at Site 4, and 20 at Site 5.

### Key Themes: Primary and Subthemes

The analysis revealed several key themes and challenges encountered by diverse Culturally and Racially Marginalized (CARM) peer workers within behavioral health systems. These findings are delineated subthemes that are seen to either support or hinder the promotion of cultural responsiveness in settings that center lived experience and Lived Expertise.

The themes include:

A. Potential enablers for CARM individuals in peer work contexts,
B. Mental health and AOD systems are being dominated by perspectives of white culture,
C. CARM people not having a voice in the peer movement,
D. Challenges faced by CARM people to take on peer work roles and within peer work contexts.

### A. Potential Enablers for CARM Individuals in Peer Work Contexts

Participants identified factors that function as potential enablers for Culturally and Racially Marginalized (CARM) people in peer work settings. These include recognizing that informal peer support began in communities, sharing a similar culture, and respecting service users’ backgrounds. Overwhelmingly, the analysis revealed more challenges faced by CARM communities in engaging in the peer workforce rather than enablers. However, it was still deemed important to center and lead with enablers, as strengths-based approaches are key to ensuring culture is not viewed solely through a deficit-focused lens.

#### A. 1. Informal Peer Support was Born in the Community Before Behavioral Health Systems

Participants described how informal peer support long served as a vital and collective means of coping and survival across generations, predating formal behavioral health systems and arising from the necessity of relying on one another for emotional support when professional care was inaccessible.

Informal peer support, supporting one another out of necessity, was part of the experience in many cultures.

I came up against a similar circumstance… with the quote unquote African American family where the perspective was that “doctors ain’t have no couches in the cotton field, you have to…suck it up”. We had that perspective that if we, for generations have been able to handle this and we should be able to do it now because what you’re facing is nothing compared to what your grandma faced in the savannah… so having the peers - that’s like what we’ve been doing all along because there wasn’t no doctor’s couch in the cotton field, we only had each other. (Site 3, FG peers)

And there’s also generational [considerations]. You know, my parents are the generation that you, pull yourself up by your bootstraps. Part of that is a religious background, and part of it is just the way they were raised. (Site 4, FG peers)

#### A. 2. Usefulness of Culturally Aligned Peer Workforces from Similar Backgrounds

In some communities, most people accessing services were from CARM backgrounds. Participants saw it as essential to have peers from CARM backgrounds, or at least to have a strong understanding and respect for the cultures within that community, through working closely and talking with traditional workers from those cultures.

I don’t think it’s about having the culture; it’s about being knowledgeable about the culture. So, I don’t necessarily think that we have to have the same exact culture in order to help somebody to be in recovery or to get them to recovery or whatever the case may be. But at least have the knowledge to be like, okay... you know, just differences, or these are the barriers that you’re looking at, you find a way to break them or do something about them. (Site 4, FG traditional workers)

But you would need that. You would need somebody of the same culture - (Site 4, FG management)

Having greater workforce representation was proposed as a way to ensure that organizations could serve more CARM people.

Now, if you want more people of color, well, then talk to your staff or your organization and say, ‘What do you think is the reason why people of color don’t come in here?’ (Site 2, INT peer manager)

### B. Behavioral Health Systems are Dominated by Perspectives of White Culture

Behavioral health services were seen to mirror the wider culture, where the leading views and positions of power represented dominant perspectives. Participants discussed cultural barriers they experienced in behavioral health settings that were created by and privileged white culture.

The subthemes under this broader theme include barriers to activism and one’s own cultural barriers in peer work, difficulties faced by folks in adjusting to clinical ways of working, the multiple levels of prejudice experienced by CARM individuals, as well as Peer leadership positions being usurped by dominant culture and what were described as ‘barely peer’ ‘white men’.

#### B. 1. Cultural Barriers to Activism and Expressing Anger

Cultural beliefs about expressing angst, injustice, and historic and current cultural oppression were seen to contribute to a situation where some Culturally and Racially Marginalized (CARM) people feel inhibited from expressing their anger/raising their voice in peer settings.

Some aspects of culture have had the rebellion suppressed out of them. Not even thinking that they have recourse to do anything about it. Culturally, they found that, ‘Hey, life has just been kicking me in the rear, and this is just another kick, that’s all it is’. (Site 3, FG peers)

Some participants reflected that the lack of understanding and acceptance of peer approaches in their cultural contexts may deter people from Culturally and Racially Marginalized (CARM) backgrounds from entering peer work.

…So, there may not be a lot of Hispanic peers, because the culture itself doesn’t allow for it. Whereas there might be more White peers… maybe it’s just our culture is more accepting of it at this point in time. …It’s not all the systemic issues of trying to prevent peers... and making it all good for the White people, but just the inherent issues within the culture. (Site 4, FG 3 peers)

#### B. 2. Clash with Clinical Culture

Participants spoke about the difficulty of integrating one’s own cultural identity into a clinical setting dominated by a different cultural framework, underscoring the clash between personal cultural values and prevailing clinical norms, which can create barriers to effective inclusion and participation.

I would find that very difficult to be a part of my culture let alone the only one from that culture in another culture because…they have their own clinical culture so that’s… my experience of that. (Site 5, FG peers)

Participants discussed how a strong peer culture could mitigate this tension and foster greater inclusion.

We’re fortunate to have a strong peer culture here, I think it would be a lot more difficult in a place where the team is more scattered and maybe there’s one peer working on each team of all a bunch of clinical people. (Site 5, FG peers)

#### B.3. CARM People Experience Multiple Levels of Prejudice

Participants emphasized the stigma experienced daily by CARM people, which influenced their perception of these environments as unwelcoming. Additionally, the complexity of navigating institutional systems where racial prejudice, gender bias, and other forms of discrimination intersect potentially hindered individuals from achieving a sense of peace or acceptance in their roles.

Honestly, I’ll just tell this as an example from my own case; it’s not an inviting place for people of color who deal with stigma every day. That’s why I think it’s more of a spiritual calling. …I think dealing with systems… I have a lot of material. I did racial healing workshops and restorative justice … so what I’ve learned is that you can find the job, but you may not be able to find peace at that job. You may not find justice at that job; you may not know how to create peace at a place where no matter what role you’re in. Cause’, believe me, there are still some people who can’t believe I’m at corporate. I have three levels of prejudice. So, I would say sometimes when people would say they want a person of color, a woman, they may not want a gay person. (Site 2, INT Peer)

#### B.4. Peer Leadership Positions are being Occupied by the Dominant Culture

Leadership positions in large organizations and health services are often occupied by those who have privilege and opportunity.

The people in power are from a very specific background [and] a very specific way of seeing things, and…the culture of the workplace is developed by them. (Site 3, FG peers)

Those in leadership positions may have little in common with those working on the frontlines. One participant identified an ‘arrogance and sense of entitlement’ among those who are given leadership positions while having little in common with clients:

If we value this idea of lived experience, it has to be intersectional; it has to also understand that to have it truly reflect people and be peer, it has to be peers. It can’t just be the white guy that went to Yale and … are actually quite privileged and have been really cushioned their whole lives from being impacted greatly by what is clearly not that serious in the wider scope of things… It’s just, it’s bananas! … when you see people who work for a year and then, they want to be managers already, and I’m like, ‘it’s a sense of entitlement …. (Site 3 INT, peer manager)

This peer manager introduced the concept of ‘barely peer’ to describe individuals who transition into peer leadership roles without having had significant life-changing experiences or having faced oppression.

They keep bringing in people who are non-peers or, I’m gonna say ‘barely peers’, because in a place like this, …suddenly people who don’t identify as peers and they didn’t, …they’ve never been hospitalized and they don’t have serious mental health concerns that have created barriers in their lives in any kind of substantive way, and over time if they see …a peer position of leadership they suddenly say, ‘yea well, I’ve always been depressed’ which may be true but I also think there’s something really interesting that’s happening here… I guess if you’re… like in [location removed], in the public mental health system most people are Black and Brown and poor, so if we’re really talking about not having an us and them, and having full representation, and you have a peer leadership position…and you have someone who doesn’t have a serious history … like I have feelings about that … I think it’s like the people who made the decisions are non-peers. (Site 3 INT, peer manager)

### C. CARM People have No Voice in the Peer Movement.

This theme included the importance of CARM communities being able to voice their opinions and the culturally prescribed standards of behavior within a workplace, reflecting the lack of voice CARM communities have in a widely ‘white’ Peer Movement.

#### C. 1. The Importance of CARM Communities to be able to Voice their Opinion

Peer participants identified that the peer movement was historically and still is dominated by white culture and that not all CARM people feel they have the right to be ‘mad’ (angry), impacting their inclusion in a peer movement dominated from inception by what were described as ‘angry white people’.

I think the traditional peer work has been made up of sort of middle-aged Caucasian people who were hospitalized that like got upset, and I’m thankful for that movement, but that hasn’t been my experience. My experience has been sort of like, I haven’t had a lot of agency over my life, so you put me in the hospital and tell me when it’s time to get out. You know, I didn’t get furious over being in the hospital. I didn’t like it, but I wasn’t furious. I didn’t have a distinct rebellion, so I think there are more voices that could be incorporated into the peer movement. (Site 3, FG peers)

Participants acknowledged the importance of giving voice to the experiences and opinions of CARM people. They spoke to the fact that within specific cultural communities, like African American and African Caribbean communities, there is often a lack of open dialogue about personal struggles and challenges. This silence on discussing sensitive topics can make individuals feel unsupported or isolated.

Letting people know that you can accomplish anything [is important], and many people don’t have an understanding of what it is because we don’t talk about these things, especially amongst cultures [like] Afro American, Afro Caribbean, we don’t discuss these things. And we don’t seek help… so for me it’s really important to have a presence and being able to stand up and to say, ‘ok this is something that I contend with’ - to give people hope, to be able to say, ‘you’re not alone’. Or, “you’re not the only ones that deal with this or have dealt with that’, so that was really important for me. (Site 3 FG peers)

#### C. 2. Prescribed Standards of Behavior in the Workplace

Participants also identified dominant perspectives within behavioral health systems dictating biased and culturally prescribed behaviors that need to be adhered to, which often exclude CARM people.

So, their [dominant perspectives’] standards are a certain way. …The rest of us who don’t fit into that find it very difficult. I know [this is] one of the things that I find very difficult to deal with as a Black woman; we believe in being direct. And the worst thing you can tell us is, ‘We’re fake.’ It’s the worst thing you can tell a Black woman, ‘You’re fake’. What, my hair might be fake, my nails might be fake, but don’t tell me, I’m fake! You know what I mean? [This has been what] My experience of working in services has been, because it’s very much the culture, is very dominated by the larger culture, [where] the focus is on diplomacy and politeness and being nice. (Site 3, FG peers)

### D. Challenges Faced by CARM People to Take on Peer Work Roles and within Peer Work Contexts

This broader theme relates to the challenges faced by CARM communities in peer work contexts, including assumptions made of CARM communities – often requiring them to confront stereotypes, challenges relating to satisfying set criteria, the role of culture in behavioral health care, as well as CARM individuals needing to work harder to prove themselves. Stigmatization of mental illness within diverse cultures is also explored.

#### D. 1. Multiple Assumptions are made of CARM People, Requiring them to Confront Stereotypes

Participants expressed challenges in recognizing intersectionality within peer support contexts and in others making assumptions based on superficial similarities, such as race or appearance, that may not accurately reflect their unique experiences.

I feel that there’s sometimes a lack of being open to the concept of intersectionality and not every person has the same exact experience just because they might kind of look like someone else and being open to that. I’ve had people make a lot of assumptions about my experiences, what I’ve been through, what I haven’t been through, demographic information, like where I grew up, how I grew up. (Site 3, FG peers)

Some peers spoke about needing to confront assumptions and being stereotyped based on their culture.

I don’t think it mattered where I was from, or my lived experience, or how I conceptualized the world that I interacted with. I think she [non-CARM colleague] was nice, and she meant well, but you know, I think it’s one of those things, being nice and meaning well isn’t enough if you don’t view people as whole people with their own experiences. You’re just kind of perpetuating more stereotypes… I think it varies from person to person. I’ve had people who, you know, belong to similar demographics to myself who didn’t ask questions and made assumptions about [other] groups of people. I think it just varies from person to person, their level of curiosity and desire to learn about others, but I would say as a woman of color in this field, I’ve definitely been able to connect more, I guess, with other folks of color. (Site 3 Peers)

#### D. 2. Rigid Criteria Create Unequal Opportunities and Barriers

Participants cited unequal employment opportunities for CARM people. This was described in the context of police checks and laws relating to working with vulnerable people, which creates barriers that exclude many people of color from employment in peer roles.

Because most of the peers are individuals that are Black and have been to jail, and you know, things of that nature. Now every peer is different, mind you, but I’m saying, speaking of being a Black peer and having worked with Black peers. There’s a law that, in the beginning, waived individuals who had past situations of going to jail or prison or whatever, that were, you know, convicted of some things. And so, they were able to get jobs working with different individuals in a community. Now you have to go through a whole situation of clearing up your name. So that kind of deterred a lot of individuals to do this work. And then every time you turned around, people, individuals, were bringing up the past. You know, if you’re in recovery and you’re seeking to better yourself, why does that have to be a slap in your face all the time? (Site 4, FG peers)

Unequal opportunities further exclude many people of CARM backgrounds from peer roles, e.g., by having peer certification exams available only in English in most places.

It was just the challenge of finding a certified peer and finding someone that was bilingual, and they never overcame that hurdle – (Site 4, FG management)

#### D. 3. The impact of Culture in Behavioral Health Care

Participants described how culture impacts mental health and broader behavioral health care. One participant described how one’s worldview influenced their mental health.

Culture’s definitely gonna play an important part of it. I mean … one of the 4 tasks of IPS (peer practice theory Intentional Peer Support) right there is worldview… so, of course, worldview is gonna play a part in…how things are. My whole perception of mental illness and how I regulate it and navigate through it is informed [by] what I know of my culture, and…what I don’t know of my culture is probably working against me, so the more you know about it, I think that helps orient you in a firmer practice of recovery and peer support. (Site 3, FG peers)

Similarly, another participant explained that, in their culture, it was crucial to first ensure that people had eaten when meeting their participant for the first time.

…the part that is really hard for me here, coming from my culture, is that you don’t have a welcoming practice for the people that we are introducing to our program. You bring ‘em in, you dump all this stuff at the nurse’s station. I told them, ‘I don’t like the nurse’s station.’ This is what I did with the people that were on my caseload: I brought them to the dining room. First thing my mum says whenever she meets new people is, ‘Are you hungry?’ Sitting and eating with people is how [you] get a relationship. (Site2, INT peer manager)

#### D. 4. CARM Individuals Need to Work Harder to Prove Themselves

Participants of CARM backgrounds spoke of needing to go above and beyond to prove themselves.

But then it goes back to being a very cultural thing, you know, to be a man of color, you have to fight that much harder, you have to go above and beyond what is expected of you. Before, if] you used to have a BA, you have opportunities for employment, but now that’s not the case. You [need to] have [post] graduate and above, so the expectation is you gotta push and push and push, so have to sacrifice … you know it’s overwhelming, it’s exhausting, it really, really is…. (Site 3, FG peers)

#### D. 5. Mental Illness is still Stigmatized within Diverse Cultures

Participants at multiple sites clarified that many cultures, including Native American, African American, African, and Hispanic, may see mental illness as highly stigmatizing. It was mentioned that instead of seeing it as an illness, many cultures believe in spiritual malaise/demons, etc. The definition of peer work as related to having a diagnosis of mental illness is therefore seen as an additional barrier contributing to the low representation of diverse cultures amongst peers.

But I think something that also needs to be paid attention to is that there is some systemic prejudice, but there’s also some cultural prejudice. But in the Hispanic culture that is very prevalent down here, mental illness is still very much looked at as something that is a curse or judgment from God, and that you just need to go and pray about it. (Site 4, FG peers)

Participants believed that a lack of peers from diverse cultural backgrounds means CARM people in the community are not receiving the most culturally appropriate support.

I know for me being part African American part native American Indian you know …we believe in shamans for our native American [side] so things related to sort of wellbeing you go to the shaman and you know he does blessings or dancing and …it’s again always a male, [whereas] amongst African American community it’s something that we would not talk about openly or publicly. (Site 3, FG Peers)

## Discussion

This study aimed to enhance the comprehension and prioritization of cultural responsiveness within peer work settings by investigating and analyzing the experiences of Culturally and Racially Marginalized (CARM) individuals from the perspectives of peer and non-peer workers, staff, and management in multidisciplinary behavioral health systems and services. The findings reveal several key themes and challenges within behavioral health systems as they pertain to CARM individuals. These findings were categorized into four main themes: the potential enablers that facilitate good practice for CARM individuals in peer work environments; the prevalence of dominating perspectives within behavioral health systems; CARM communities not having a voice in the peer movement; and the difficulties encountered by CARM individuals in peer work roles and contexts.

Revitalizing community-led informal peer support as a potential enabler. The findings highlight several potential enablers that may inform more equitable and sustainable approaches to engaging CARM individuals in peer work contexts, alongside the numerous challenges reported. Informal peer support has long served as a vital means of coping and survival across generations—emerging within grassroots communities, such as African American communities, out of necessity when professional care was not accessible. This has been widely recognized across the peer movement, underpinned by peer support’s prevailing identity as a “grassroots form of social support” (Stratford et al., 2019). Although these traditional and organic forms of support have often been eroded through the shift to centralized systems dominated by predominantly white cultural norms, their historical role underscores a critical potential enabler: the recognition, revitalization, and integration of community-led informal peer support within contemporary behavioral health systems.

### Building a Culturally Relevant and Diverse Peer Workforce

In communities where the majority of service users are from CARM backgrounds, having peers who share these backgrounds or possess a profound understanding of the prevalent cultures is crucial. Close collaboration with traditional workers from these cultural backgrounds facilitated this understanding and aligned with the literature, which states they may be better positioned to “recognize local needs and resources, to understand local culture, to select and adapt appropriate… practices, and to innovate solutions” (Drake & Whitley, 2015). Addressing the lack of diversity in the workforce is a proactive step organizations can take to improve outreach and support for more CARM communities.

### Navigating Dominant Power Systems and Cultural Expressions of Resistance

The dominance of traditional white perspectives within mental health systems was also a pervasive theme reported by participants. Cultural beliefs regarding the expression of distress and experiences of historical and ongoing marginalization contribute to scenarios where CARM individuals may feel inhibited from voicing anger or asserting themselves within activism in peer environments. This may be viewed as ironic, given that anger has been seen to serve as a “strategy for survival” and a “means of resistance” for Black and indigenous communities (Bargallie, 2018), as well as an appropriate response to injustice (Lorde, 1981). Challenges regarding cultural comprehension and acceptance from White peers within these settings were also highlighted.

### Challenging Medical Model Dominance and Narrow “Evidence” Norms

Services often reflect broader cultural norms, with dominant perspectives prevailing and mirroring the wider culture. This aligns with the broader literature, which describes the dominant discourse as aligned with the "medical or clinical model," which often overlooks social, structural, and cultural factors that influence mental health and well-being (Corneau & Stergiopoulos, 2012). This is further aligned with concepts such as "Evidence-based practice" (EBP), which, although historically regarded as universally applicable, are increasingly being critiqued for their "epistemological narrowness and exclusion of communities of color” (Aisenberg, 2008).

Intersectional harms, organizational bias, and privileged leadership in peer work. The difficulties experienced by CARM individuals encompass multiple dimensions, including intersectionality, systemic prejudice, cultural integration, and leadership roles.

The compounded prejudices based on race, gender, and other factors create inhospitable environments for CARM community members, impacting their sense of inclusion and effective participation in clinical settings.

In relation to how this translates to challenges for CARM individuals within peer work roles and contexts, recognizing the importance of intersectionality within peer support contexts was also crucial, given that assumptions based on race or appearance often fail to capture the diverse experiences of individuals, leading to stereotyping and a superficial understanding of their backgrounds, thus impacting their professional lives. Riggs and das Nair R. (2012) further draw attention to this notion, stating that simply "adding on more identities (whilst treating them all as separable)" fails to recognize the complexities of people’s lives. Organizational values often do not align with cultural perspectives, leading to implicit organizational biases (Desai et al., 2021). Leadership positions within organizations frequently reflect privilege and fewer life disruptions, not genuinely representing marginalized communities. This is especially pertinent given that a supportive supervisor attitude has been noted as critical for establishing a facilitative environment for peer support specialists (Forbes et al., 2022). Participants also noted an "arrogance and sense of entitlement" among those who have not faced significant life disruptions, emphasizing the need for diversity and cultural representation in leadership roles. There is also emerging discourse that addresses the tensions between current leaders in the peer work space and diverse approaches, noting that individualistic leadership practices can conflict with collective action principles and are often sustained by unacknowledged relative power (e.g., whiteness, heterosexuality, able bodied-ness) among leaders who, despite experiencing other forms of marginalization, occupy dominant social positions (Higgins and Lenette, 2024). This could lead to a valid sense of injustice, which may inhibit reflexivity, highlighting the need for this existing leadership to acknowledge their relative privilege, particularly in periods of reform.

### Amplifying CARM Voice and Reshaping Peer Norms Beyond Individualism

Providing a platform for CARM communities to articulate their viewpoints is essential, given the prevailing epistemic injustice that seeks to maintain dominant knowledge systems (Hammond, 2025). The peer movement, historically and currently dominated by white culture, continuously reflects "social formations of domination being produced within a broader system of white supremacy" (Joseph, 2019). Some CARM individuals feel they lack the right to express anger, which, for some, has affected their ability to assert themselves within a peer movement built on principles of advocacy, agency, and empowerment. This dynamic also reflects the concept of ‘microinvalidation,’ which has significant implications for both the perpetrator and the target (Sue et al., 2007).

The prevailing viewpoints within behavioral health systems establish norms of behavior that must be followed, often resulting in the exclusion of CARM individuals. Evidence from the peer literature indicates that peer leaders from collectivist and family-centered cultures find the emphasis on individual recovery, self-determination, and agency to be foreign to their way of thinking (Stratford et al., 2019). This may pose significant barriers to engagement, leading to an underrepresentation of diverse cultural perspectives among peers in the peer workforce. However, it may be beneficial to explore what a broader mutual learning relationship can look like, given the breadth of literature that speaks to the benefits of integrating culture in peer work contexts across a range of disciplines and areas (Cooeyate et al., 2024; Edwards & Solomon, 2023; Fang et al., 2024; Hodges, 2023; Usideme, 2024).

### Addressing Systemic Employment Barriers and Recognition gaps for CARM Peers

Challenges faced by CARM individuals in peer-work roles were another significant concern. Disparities in employment opportunities and barriers to career advancement play a significant role, excluding many CARM individuals despite possessing relevant life experiences and a desire to work as peers. Many CARM individuals face systemic challenges such as criminal records, police checks, and laws governing work with vulnerable populations, which preclude them from peer employment, despite literature more broadly recognizing that an individual’s intersecting identities can create “disproportionate outcomes for both agents and consumers of criminal justice” (Workman et al., 2023). Additionally, peer certification exams, which are primarily available in English, further exclude non-English-speaking CARM individuals from obtaining peer roles.

CARM individuals often also feel they must exceed expectations to be validated in peer work settings. There is a dearth of literature on this in the peer work context, underscoring the need for further research. However, this notion is well documented in fields that align, such as bicultural work, which notes that CARM individuals must often work extra hard to explain their value, expertise, and credibility to colleagues whilst continuing to experience disregard in several ways (McDonough, 2020). Consequently, the lack of diversity among peers results in inadequate structural and practical support for diverse community members, despite local community responses often “producing positive changes and sometimes leading to national and international health reforms” (Drake & Whitley, 2015).

In conclusion, addressing the challenges faced by CARM peer workers in behavioral health systems requires integrating cultural humility (Foronda et al., 2015), active intersectionality, and genuine valuing and inclusion within these systems. By recognizing and addressing systemic biases and cultural barriers, behavioral health systems can move towards more inclusive, responsive, and revolutionary practices that genuinely reflect the intersectionality of the communities they serve.

### Implications and Recommendations

The findings of this study have significant implications for practice, underscoring the need for systemic and structural changes to ensure peer work environments are inclusive, equitable, and reflective of the diverse, intersectional communities they serve.

Recommendations from this study include:

1. It is necessary for organizations to examine the dominance of traditional perspectives within behavioral health systems thoroughly. This requires a critical reassessment of existing frameworks through a nuanced lens, such as addressing cultural diversity in "evidence-based practices" (EBP) whilst grounding cultural responsiveness work in evidence (Kirmayer, 2012), to better incorporate culture-oriented lived expertise.
2. Training and vocational programs and policies need to be assessed through relevant cultural guidelines such as the ‘Framework for Mental Health in Multicultural Australia’ (2026) or the work of the Multicultural Affinity Group of Australia’s consumer peak body, National Mental Health Consumer Alliance (The Alliance) (2026). This is to embed more inclusive methodologies that acknowledge the unique structural, cultural, and social drivers of mental well-being for Culturally and Racially Marginalized (CARM) people, which have long been shown to reduce healthcare disparities (Williams et al., 2008).
3. Addressing existing systemic privileges and biases within peer work environments is also integral to ensuring that CARM peer workers can participate authentically without feeling invalidated or marginalized. In practice, this may involve examining how, for example, the *Intertwine Charter* (2026) can be operationalized in the workplace to identify proactive organizational strategies to address the ways intersecting oppression, privilege, and resistance may manifest.
4. Identifying and addressing the barriers that limit genuine CARM engagement in peer support roles actively. This includes reworking hiring practices and procedures as well as addressing obstacles (such as criminal background checks) that may disproportionately affect individuals from CARM communities. Additionally, increasing educational and financial accessibility for CARM communities, as in the approach taken by Mental Health Victoria, AIDMH’s Solis Network and the Victorian Department of Health’s *Strengthening Intersectionality and Diversity of the Lived and Living Experience Workforce (SID-LLEW)* grants program (2025), can intentionally sustain the development of CARM peer workers. Expanding training opportunities and peer certification in various languages and culturally responsive formats will also facilitate greater inclusivity within peer workforce contexts.
5. There is a pressing need to increase diverse and intersectional leadership within behavioral health services. For CARM communities to feel supported and empowered, there must be representation at the highest levels of decision-making and governance, as this has been explicitly linked to positively impacting health and well-being outcomes across a range of settings (Bradley, 2020; Zambrano, 2019).
6. To ensure that organizational cultures shift toward meaningful inclusion rather than tokenistic representation, tailored development programs should be introduced to uplift CARM voices in peer work settings, including early pipeline models such as The University of New Mexico’s Dream Makers Health Career Programs that partner with schools and colleges to qualify low-income CARM students, while strengthening and diversifying the broader behavioral University of New Mexico (2026).

### Limitations and Further Research

This paper was primarily written by authors from a Culturally and Racially Marginalized (CARM) background, with knowledge and experience in peer roles. Another strength of the study is its substantial sample size of 132 key participants for qualitative research. However, it is limited to organizations within the United States, which may restrict the study’s transferability to other countries and cultures. Despite these limitations, the data provides important insights and recommendations for organizations to increase diversity and inclusion for CARM workers and adds to the limited existing literature on the topic.

Further research is needed to establish credibility beyond the United States and to gain confirmation from an international perspective. More targeted research is also needed to investigate the intersection of culture, race, and peer work, and the potential impacts on the accessibility and effectiveness of peer support services across cultures. Furthermore, research should explore the strategies that organizations have successfully employed to foster a genuine sense of inclusion, while identifying best practices for supporting CARM individuals in these roles. An additional avenue for future research is exploring the emotional and psychological toll of tokenism, microaggressions, and other exclusionary practices in relation to CARM people in peer work settings. Exploring existing resilience strategies, coping mechanisms, and organizational supports to such challenges can inform policies and procedures that better uphold the well-being of CARM peer workers.

## Conclusion

In conclusion, addressing the challenges faced by CARM peer workers in behavioral health systems requires integrating cultural responsiveness, intersectionality, and genuine valuing of inclusion within these systems. By recognizing and addressing systemic biases, organizational barriers to embedding culture, and personal experiences, behavioral health systems can move towards more inclusive and responsive practices that meaningfully reflect the diversity and strengths of the communities they serve.

## Competing Interests

The authors declare no competing interests.

## Data Availability

The data that support the findings of this study are not openly available due to reasons of sensitivity and are available from the corresponding author upon reasonable request.

## Appendix 1: Nomenclature. Defining Culturally Diverse Communities – CALD, BIPOC, CARM etc.

The authors gave careful consideration, within their academic, social, and professional networks, to the language used to refer to the communities and demographics central to this research. It is crucial to understand and critically examine the purpose behind the use of labels by communities, organizations, and policymakers. This has been demonstrated in several community-led initiatives, for example, in the Victorian State context in Australia (AIDMH, 2023). While categorizing individuals based on demographic characteristics can be useful and sometimes necessary, it should seek to facilitate understanding, thereby informing conceptualization and design of more effective services, supports, or programs that genuinely respond to the needs of communities, rather than simply labelling them.

We considered several widely used terms in this process, including their pros and cons, as illustrated in Table 1 below. Despite the evolution of language and numerous perspectives on this matter, our primary goal is to encourage a reflective and intentional approach to these complex issues. After deliberation, we have chosen to use the term "culturally and racially marginalized" (CARM) to refer to the peer workers who are the focus of this research. We acknowledge their experiences within the broader context of marginalization. However, we do this with a caveat: although we address a subset of challenges in this paper, the vast and boundless strengths of cultural backgrounds cannot be understated and should, alongside meaningful lived-experience representation, drive our work.

**Table 1:** Defining Culturally Diverse Communities – CALD, BIPOC, CARM

Term	CALD (Culturally and Linguistically Diverse)	BIPOC (Black, Indigenous, People of Color)	CARM (Culturally and Racially Marginalized)
**Pros**	Widely known in the Australian context, frequently used in government and sector discussions – can make it easier to advocate	Well-known and frequently used in the North American context, recognizes that people of color face different challenges and centers Black and Indigenous experiences which can be valuable as a way of thinking, given how violence against Black and Indigenous people is foundational to the United States, a country founded on the enslavement of Black people and the genocide of Indigenous people	In Australia, this term is emerging as a way to refer to non-white groups who face marginalization due to race or culture. It acknowledges the social processes of racialization and the unequal treatment that results
	Overly simplistic, as it applies to everyone to some degree, and fails to address how cultural factors can specifically impact an individual’s experience. At times is confusing and may be used incorrectly by white people to describe themselves	Risks imposing a US-centric perspective on contemporary, global racial issues, potentially oversimplifying diverse groups and experiences within other countries Not always a preferred term for the people whom it is designed to describe	In some contexts, the term may unintentionally emphasize deficits rather than the strengths that cultural and racial diversity bring to the table. A strengths-based perspective alongside it’s use is necessary to ensure that cultural identity is rightfully centred as a positive force in people’s lives
